# Optimization of conditions for *in vitro* modeling of subgingival normobiosis and dysbiosis

**DOI:** 10.3389/fmicb.2022.1031029

**Published:** 2022-11-03

**Authors:** Divyashri Baraniya, Thuy Do, Tsute Chen, Jasim M. Albandar, Susan M. Chialastri, Deirdre A. Devine, Philip D. Marsh, Nezar N. Al-Hebshi

**Affiliations:** ^1^Oral Microbiome Research Laboratory, Maurice H. Kornberg School of Dentistry, Temple University, Philadelphia, PA, United States; ^2^Division of Oral Biology, School of Dentistry, University of Leeds, Leeds, United Kingdom; ^3^Department of Microbiology, Forsyth Institute, Cambridge, MA, United States; ^4^Department of Periodontology and Oral Implantology, Maurice H. Kornberg School of Dentistry, Temple University, Philadelphia, PA, United States

**Keywords:** biofilm, dysbiosis, high-throughput nucleotide sequencing, microbiota, periodontitis

## Abstract

Modeling subgingival microbiome in health and disease is key to identifying the drivers of dysbiosis and to studying microbiome modulation. Here, we optimize growth conditions of our previously described *in vitro* subgingival microbiome model. Subgingival plaque samples from healthy and periodontitis subjects were used as inocula to grow normobiotic and dysbiotic microbiomes in MBEC assay plates. Saliva supplemented with 1%, 2%, 3.5%, or 5% (v/v) heat-inactivated human serum was used as a growth medium under shaking or non-shaking conditions. The microbiomes were harvested at 4, 7, 10 or 13 days of growth (384 microbiomes in total) and analyzed by 16S rRNA gene sequencing. Biomass significantly increased as a function of serum concentration and incubation period. Independent of growth conditions, the health- and periodontitis-derived microbiomes clustered separately with their respective inocula. Species richness/diversity slightly increased with time but was adversely affected by higher serum concentrations especially in the periodontitis-derived microbiomes. Microbial dysbiosis increased with time and serum concentration. *Porphyromonas* and *Alloprevotella* were substantially enriched in higher serum concentrations at the expense of *Streptococcus*, *Fusobacterium* and *Prevotella*. An increase in *Porphyromonas*, *Bacteroides* and *Mogibacterium* accompanied by a decrease in *Prevotella*, *Catonella,* and *Gemella* were the most prominent changes over time. Shaking had only minor effects. Overall, the health-derived microbiomes grown for 4 days in 1% serum, and periodontitis-derived microbiomes grown for 7 days in 3.5%–5% serum were the most similar to the respective inocula. In conclusion, normobiotic and dysbiostic subgingival microbiomes can be grown reproducibly in saliva supplemented with serum, but time and serum concentration need to be adjusted differently for the health and periodontitis-derived microbiomes to maximize similarity to *in vivo* inocula. The optimized model could be used to identify drivers of dysbiosis, and to evaluate interventions such as microbiome modulators.

## Introduction

The oral microbiome is complex and diverse, but remains balanced over time (normobiosis), generally existing in a harmonious and mutually beneficial relationship with the host. However, this mutualistic relationship can breakdown due to changes in the balance of the microbiome and/or to the integrity of the host defenses (dysbiosis), and this increases the risk of disease (Kilian et al., 2016). The wide scale application of next generation sequencing technologies has revolutionized our understanding of the composition of the oral microbiome in health and disease. In periodontitis, dysbiosis is associated with increases in predominantly anaerobic, Gram-negative bacteria including *Treponema* spp., *Fretibacterium* spp., *Prophyromonas gingivalis*, *Tannerella forsythia*, and *Desulfobulbus* spp. at the expense of facultative Gram-positive species belonging to the genera *Actinomyces* and *Streptococcus* (Curtis et al., 2000; Chen et al., 2022).

However, the drivers of subgingival microbial dysbiosis, which could make novel targets for interventions, are still not well understood. Likewise, the potential for microbiome modulators, such as prebiotics and probiotics, to reverse dysbiosis or maintain normobiosis as a treatment and/or prevention strategy for periodontitis has been minimally explored. Addressing these gaps in clinical studies is challenging due to the wide range of variables that can affect the oral microbiome. Potential interventions need to be tested in a preclinical environment, and therefore, validated *in vitro* microbiome models are important research tools in this respect. Various attempts have been made to model oral microbial communities including continuous flow models, static models or more recent sophisticated models that are based on microfluidics or impedance-based technologies (Brown et al., 2019). Over the last 20 years, some static biofilm models (e.g., microtiter plates or the Calgary Biofilm Device) have become popular due to their ease of performance and high throughput (Brown et al., 2019). Combined with the use of saliva or oral biofilms as inocula, these static models have been used successfully to generate highly diverse *in vitro* microbiomes that approximate to the complexity and community structure of clinical samples (Walker and Sedlacek, 2007; Tian et al., 2010; Edlund et al., 2013; Kistler et al., 2015; Kolderman et al., 2015; Baraniya et al., 2020).

We recently developed an *in vitro* model of the subgingival microbiome in which health- and periodontitis-derived microbiomes are grown in parallel in a high throughput format (Baraniya et al., 2020), which can be used to study the dynamics and drivers of subgingival dysbiosis and, more importantly, to screen for microbiome modulators especially when combined with our recently described subgingival microbial dysbiosis index (Chen et al., 2022). In this model, we found that saliva supplemented with 5%–20% (v/v) heat-inactivated human serum outperformed nutrient rich media, including BHI (Brain Heart Infusion) and SHI, in terms of maintaining viability of the biofilms, maximizing species diversity, and replicating normobiosis and dysbiosis. Nevertheless, it was observed that these relatively high serum concentrations led to the enrichment of *P. gingivalis* in a dose-dependent manner which tended to increase dysbiosis scores specially in the health-derived microbiomes.

In this study, we further explored the nutritional drivers of subgingival dysbiosis by lowering serum concentration, varying the incubation periods and evaluating the impact of shaking, with the aim of maximizing similarity to *in vivo* microbiomes. By running two biological replicates, we also aimed to demonstrate the reproducibility and utility of the model in generating subgingival normobiotic and dysbiotic microbiomes.

## Materials and methods

### Study design and ethics statement

**Figure 1:**
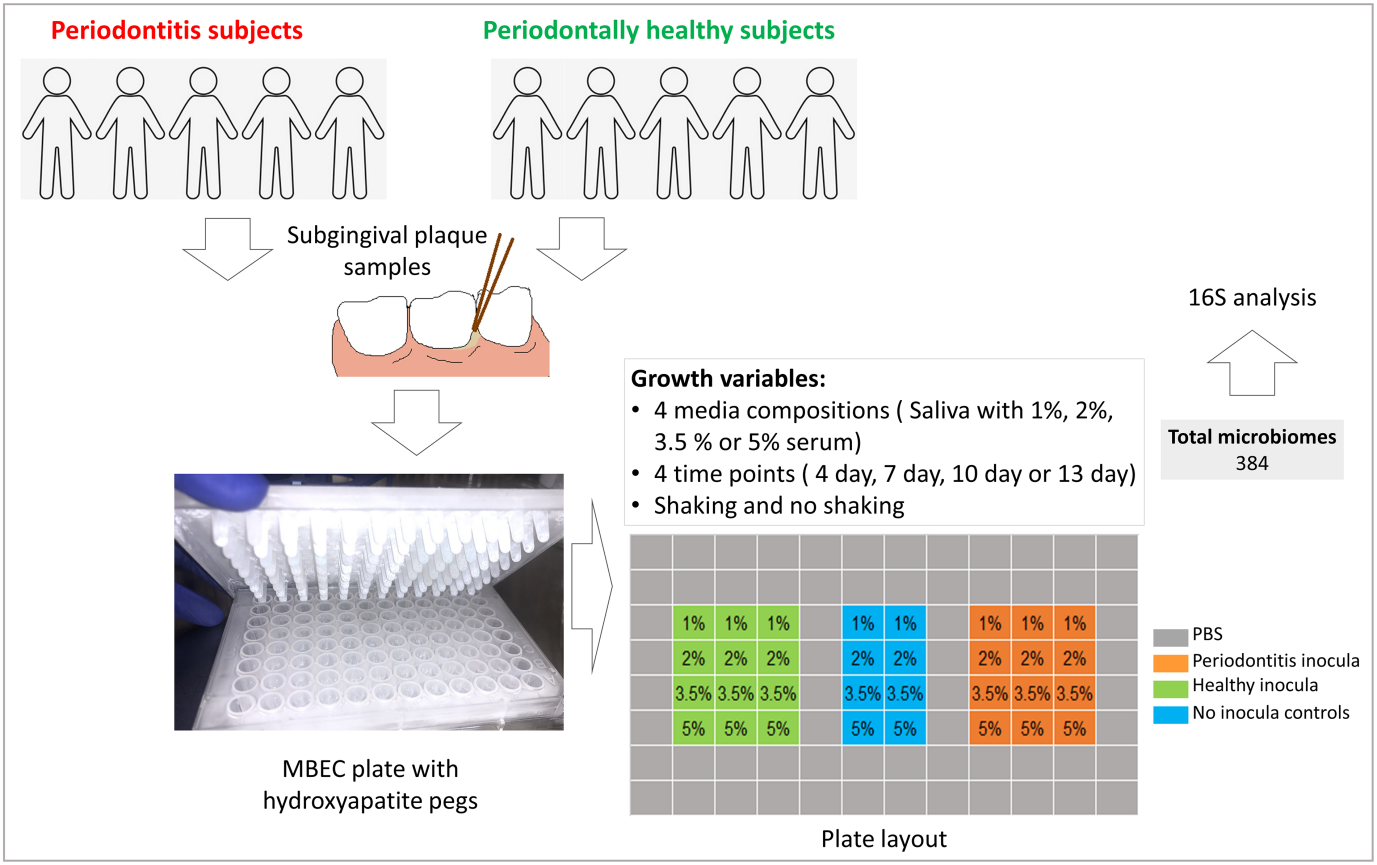
A flow chart of study design and procedures.

Figure 1 provides an overview of study design and workflow. Subgingival dental plaque samples were collected from patients that were being treated at the Dental Clinics of the Kornberg School of Dentistry, Department of Periodontics, Temple University, Philadelphia, United States, after obtaining informed consents. The study was approved by Temple University’s Institutional Review Board under protocol #25586.

### Sterile pooled human saliva

Unstimulated saliva was collected in sterile containers from 10 dentally healthy volunteers, who had no severe cavities, no more than slight, localized gingivitis, no pocket depth or attachment loss ≥ 3 mm, and no history of periodontitis. Additionally, participants were asked to not consume food, juice, or caffeinated drinks within 2 h prior to donating saliva samples. The samples were placed on ice immediately after collection, and transferred to the laboratory where they were pooled, centrifuged at 5,000 g for 10 min, treated with dithiothreitol at 2.5 mM, filter sterilized, and stored at −20°C until used in the study.

### Clinical inocula

For each experiment, subgingival plaque samples were collected from five subjects with moderate to severe periodontitis (defined as having at least one tooth per quadrant with bleeding on probing, pocket depth ≥ 5 mm and attachment loss ≥ 4 mm) and from five periodontally healthy subjects (defined as having no more than slight gingivitis, no probing pocket depth or attachment loss ≥ 3 mm and no previous history of periodontitis). The samples were collected using paper points and placed in 1 ml of reduced transport fluid (RTF) as previously described (Baraniya et al., 2020). The two sets of samples, one from healthy subjects and the other from periodontitis individuals, were pooled separately and mixed by vortexing, yielding two homogenous suspensions which were used as a final inoculum for health- and periodontitis-derived biofilms, respectively. Samples were obtained from a completely different set of subjects for the two experiments (biological replicates)—Supplementary Table 1.

### Culture media

Heat inactivated human serum (Sigma, United States) was added to Sterile pooled human saliva (SPHS) prepared as described above at final concentrations of 1%, 2%, 3.5%, and 5% (v/v) to obtain a total of four different growth media.

### Growth of *in vitro* microbiomes

Health- and periodontitis-derived microbiomes were grown on hydroxyapatite-coated pegs of MBEC plates (Innovotech, Edmonton, Canada) as described before (Baraniya et al., 2020). Briefly, the pegs were preconditioned by immersing in SPHS for 16 h before they were transferred to a 96 well plate containing 170 μl culture medium plus 10 μl inoculum per well in triplicates according to the layout shown in Figure 1; the outer wells were filled with sterile PBS to prevent evaporation. No-inoculum control wells were spiked with 10 μl sterile RTF. The microbiomes were grown for 4, 7, 10, or 13 days (four different plates) under shaking or non-shaking conditions (two sets of plates). Sample handling, plate preparation, inoculation and incubation were all performed inside an anaerobic chamber supplied with a gas mixture comprising 10% hydrogen, 10% carbon dioxide, and 80% nitrogen. The pegs with microbiomes were harvested at the end of each time point, washed with PBS for 15 s and immediately stored at −80°C for further analysis. The experiment was performed twice with two different sets of clinical inocula (biological replicates), yielding a total of 384 *in vitro* microbiomes.

### DNA extraction and biomass assessment

Pegs with the grown microbiomes were each snapped off from the lid, placed in 1.5 ml tubes containing 180 μl lysozyme solution (20 mg/ml; Sigma, United States) and incubated for 30 min at 37°C. DNA was extracted from the lysate using Purelink Genomic DNA mini Kit (Invitrogen, Waltham, United States) according to the manufacturer’s instructions. DNA was quantified using Qubit 2.0 Fluorimeter and stored at −80°C until subjected to further analysis. Biomass was measured in terms of DNA yield (ng/microbiome).

### Sequencing and data analysis

Degenerate primers 27FYM (Frank et al., 2008) and 519R (Lane et al., 1985) with index sequences were used for amplifying the V1–V3 region of the 16S rRNA gene, and the resultant indexed amplicon libraries were sequenced on an Illumina Miseq platform using 2*300 bp chemistry at the Integrated Microbiome Resource (IMR, Halifax, Canada). Resultant paired-end reads were merged with PEAR (Zhang et al., 2014) and pre-processed (trimming, quality filtration, and chimera check) with mothur (Schloss et al., 2009) as previously described (Al-Hebshi et al., 2017). The high-quality reads were then classified to the species level using our previously described BLASTn-based algorithm (Al-Hebshi et al., 2015, 2017). Taxonomy tables and alpha diversity analysis were generated using Quantitative Insights into Microbial Ecology (QIIME2; Caporaso et al., 2010). The Subgingival Microbial Dysbiosis Index (SMDI) was calculated for the individual samples based on abundances of 49 discriminating species as previously described (Chen et al., 2022). For assessing beta diversity, principal component analysis (PCA) was performed on centered log-ratio (CLR) species counts using microbiome (Leo Lahti, 2017) and phyloseq (McMurdie and Holmes, 2013) packages in R. MaAsLin2 (Mallick et al., 2021) was applied to CLR-transformed data to identify independent associations with each of the study variables (serum concentration, time points and shaking). Optimal growth conditions were defined as those that maximized similarity of the *in vitro* microbiomes to the respective inocula in terms of species richness, alpha and beta diversity and SMDI values.

## Results

### Sequencing and data preprocessing statistics

A total of 26,208,640 paired-end reads were obtained (31,804 to 1,23,233 reads/sample), of which 95.24% reads merged successfully. Around 17% (4,310,661) of the sequences passed our stringent quality filtration, resulting in a final sequencing depth of 4,681 to 20,450 reads/sample. Raw sequences were submitted to Sequence Read Archive (SRA) under project no. PRJNA881921.

### General microbial profiles of the inocula and *in vitro* microbiomes

A total of 119 and 137 species belonging to 39 and 40 genera and 8 phyla were detected in the two health inocula, respectively; in the two periodontitis inocula, 178 and 172 species, 55 and 65 genera, and 9 phyla were identified, respectively. In the *in vitro*-grown microbiomes, the comparable profiles were 58–103 species, 20–33 genera, 4–7 phyla in the health-derived microbiomes, and 75–135 species, 32–52 genera and 6–8 phyla in the periodontitis-derived microbiomes. The relative abundances and detection frequencies of identified phyla, genera and species in each of the samples are listed in Supplementary Files 1–3, respectively, while the average taxonomic profiles in the inocula as well as *in vitro* microbiomes as a function of serum concentration, incubation period and shaking are presented in Supplementary Figures 1, 2, for the phylum- and genus-level, respectively.

Firmicutes, Fusobacterium, and Bacteroidetes, in this order of abundance, were the most dominant phyla in health in both the clinical inocula as well as *in vitro* microbiomes. The same phyla were also the most abundant in periodontitis but in the order of Bacteroidetes, Firmicutes and Fusobacterium by abundance. However, in both health and periodontitis, these three phyla were over-represented in the *in vitro* microbiomes at the expense of the Saccharibacteria, Proteobacteria, and Actinobacteria (Supplementary Figure 3). Chloroflexi was exclusively detected in the periodontitis inoculum but not in the respective *in vitro* microbiomes. At the genus level, *Fusobacterium*, *Streptococcus*, *Porphyromonas*, *Prevotella*, and *Alloprevotella* were the most abundant overall, although their relative abundances differed between health and disease and between the clinical inocula and the *in vitro* microbiomes (Supplementary Figure 2). The latter showed enrichment of *Fusobacterium* and *Prevotella* in addition to *Mogibacterium, Catonella*, and *Bacteroides* at the expense of *Leptotrichia*, *Rothia*, *Haemophilus*, *Capnocytophaga*, and TM7 genera 1 and 5 (Supplementary Figure 3).

At the species level, the dominant species in the health-derived microbiomes on average were *Fusobacterium periodonticum, Fusobacterium nucleatum, Streptococcus dentisani, Mogibacterium diversum, Porphyromonas endodontalis, Alloprevotella tannerae, Porphyromonas* oral taxon 278*, Prevotella intermedia, Streptococcus* oral taxon058*, Catonella morbi,* and *Veillonella parvula_group,* while periodontitis derived microbiomes were dominated by *Prevotella intermedia, Porphyromonas gingivalis, Bacteroides heparinolyticus, Bacteroides zoogleoformans, Fusobacterium nucleatum, Fusobacterium periodonticum, Streptococcus tigurinus,* and *Peptoniphilaceae* oral taxon 790.

### *In vitro* microbiomes replicate subgingival normobiosis and dysbiosis

**Figure 2:**
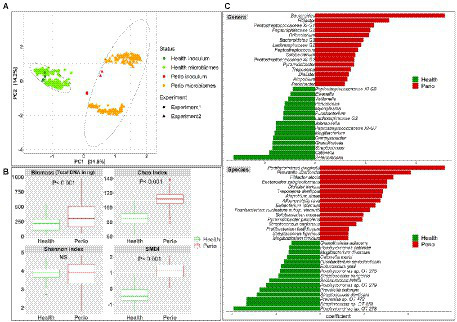
*In vitro* microbiomes from health and periodontitis. **(A)** Principal component analysis (PCA) plots based on centered log-ratio (CLR) transformed data as a function of health status and experimental replicates. **(B)** Boxplots of biomass, alpha diversity indices, and subgingival microbial dysbiosis index (SMDI) in the health- and periodontitis-derived microbiomes. Statistical comparisons were performed using Mann–Whitney test. **(C)** Top differentially abundant genera and species between the health- and periodontitis-derived microbiomes with FDR value < 0.1 and coefficient > 2 or < −2 (MaAsLin2 analysis on CLR transformed data with health status, biological replicate, medium, shaking and incubation time as fixed effects and technical replicates as random effects). Plots were produced with R.

Regardless of growth conditions, the health- and periodontitis-derived microbiomes along with the respective clinical inocula formed two separate main clusters in beta diversity analysis, accounting for ~32% variation along principal component 1 (Figure 2A)—the biological replicates formed sub-clusters within each cluster and accounted for less variation (14% along principal component 2), primarily in periodontitis. Similarly, the health- and periodontitis-derived microbiomes reflected the differences between the respective inocula in terms of biomass, species richness (Chao index), alpha diversity (Shannon index) and dysbiosis (SMDI; Figure 2B), with all being significantly higher (with the exception of Shannon index) in the periodontitis-derived microbiomes.

**Figure 3:**
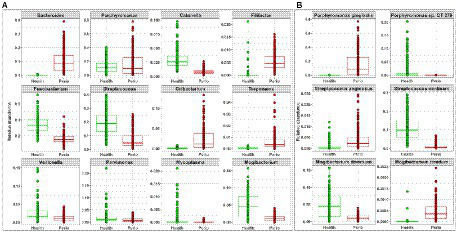
Selected differentially abundant taxa in the periodontitis and health-derived microbiomes. Centered log-ratio (CLR) transformed data were analyzed with MaAsLin2 including health status, biological replicate, medium, shaking, and incubation time as fixed effects and technical replicates as random effects. Relative abundances are shown for **(A)** 12 genera and **(B)** 3 pairs of “sister” species that significantly differed (FDR value < 0.1) between the two groups. Plots were produced with R.

More importantly, differential abundance analysis by MaAsLin2 identified microbial differences between the health- and periodontitis-derived *in vitro* microbiomes that are largely consistent with known differences between periodontitis and health *in vivo* (Figure 2C). For example, *P. gingivalis*, *P. intermedia*, *Treponema denticola*, *Filifactor alocis*, *Fretibacterium fastidiosum*, *Pyramidobacter piscolens*, and *Mogibacterium timidum*, which have been consistently implicated as pathogens in periodontitis, were all significantly enriched in the periodontitis-derived microbiomes. Likewise, species such as *Porphyromonas catoniae*, *Streptococcus dentisani, S. sanguinis*, *Streptococcus* oral taxon 58, *Catonella morbi* and *Granulicatella adiacens,* which are typically health-associated species, were significantly enriched in the health-derived microbiomes. Figure 3 presents the relative abundances of selected differentially abundant genera and species. The latter were chosen to represent sister species (i.e., two species within the same genus) that showed opposite enrichment in the health- and periodontitis-derived microbiomes consistent with differences demonstrated in their preponderance *in vivo*.

### Higher serum concentration and longer incubation time promote dysbiosis

**Figure 4:**
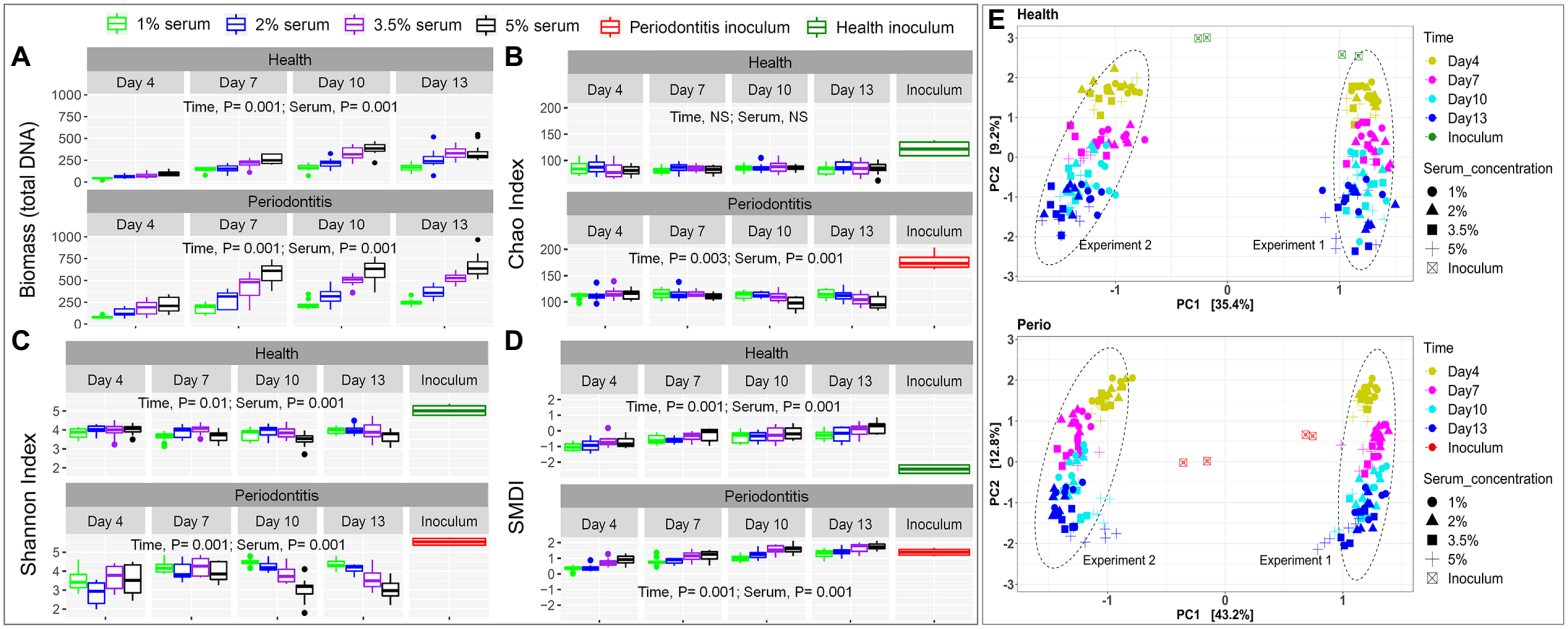
*In vitro* microbiome diversity as a function of serum concentration and incubation period. Biomass **(A)**, Chao Index **(B)**, Shannon Index **(C)**, and subgingival microbial dysbiosis index (SMDI, **D**) are presented as boxplots by serum concentration and time point for the health- and periodontitis-derived microbiomes separately. Statistical comparisons were performed using generalized linear models (multinomial distribution) with biological replicate, medium, shaking and time as covariates. **(E)** Principal component analysis (PCA) plots based on centered log-ratio (CLR) transformed data by serum concentration, time point and experimental replicate. Plots were produced with R.

Generalized linear modeling or MaAslin2, as appropriate, were used to identify the independent effects of serum and incubation time on the different microbial parameters assessed, applying a false discovery rate (FDR) cutoff of 0.1 when applicable. Biofilm biomass significantly increased with time and with increasing serum concentrations for both the health- and periodontitis-derived microbiomes (Figure 4A). Species richness (Chao index) did not change by time and serum concentration in the health-derived microbiomes, but it significantly dropped in 5% serum and after days 10 and 13 incubation in the periodontitis-derived microbiomes (Figure 4B). Alpha diversity (Shannon index) significantly decreased in days 7 and 10, increased at 2% and 3.5% serum but dropped at 5% in the health-derived microbiomes; however, the magnitude of changes was small (Figure 4C). In the periodontitis-derived microbiome, the Shannon index substantially increased with time but markedly decreased as a function of serum concentration. Dysbiosis (SMDI) increased proportionally as a function of time and serum concentration, in both the health-derived and periodontitis-derived microbiomes grown *in vitro* (Figure 4D), being closest to the respective clinical inocula in the health-derived-microbiomes when grown in 1% serum for 4 days (Median SMDI of −1 in the *in vitro* microbiomes compared to −2.2 in the health inoculum), and in the periodontitis-derived-microbiomes after growth in 1% serum for 13 days (Median SMDI of 1.33 in the microbiomes compared to 1.38 in the periodontitis inoculum); those grown in 3.5%–5% for 7 days or 2% for 10 days came next (SMDI ~1.25). Beta diversity analysis for health and periodontitis separately resulted in two main clusters by biological replicate along PC1 and sub-clusters by growth time along PC2 (Figure 4E); analysis of Aitchison’s distances revealed that the health-derived microbiomes grown for 4 days at 1% serum concentration and the periodontitis-derived microbiomes grown for 4 days in 5% serum (followed by those grown in 5% for 7 days or 2% for 10 days) were the closest to the respective clinical inocula.

**Figure 5:**
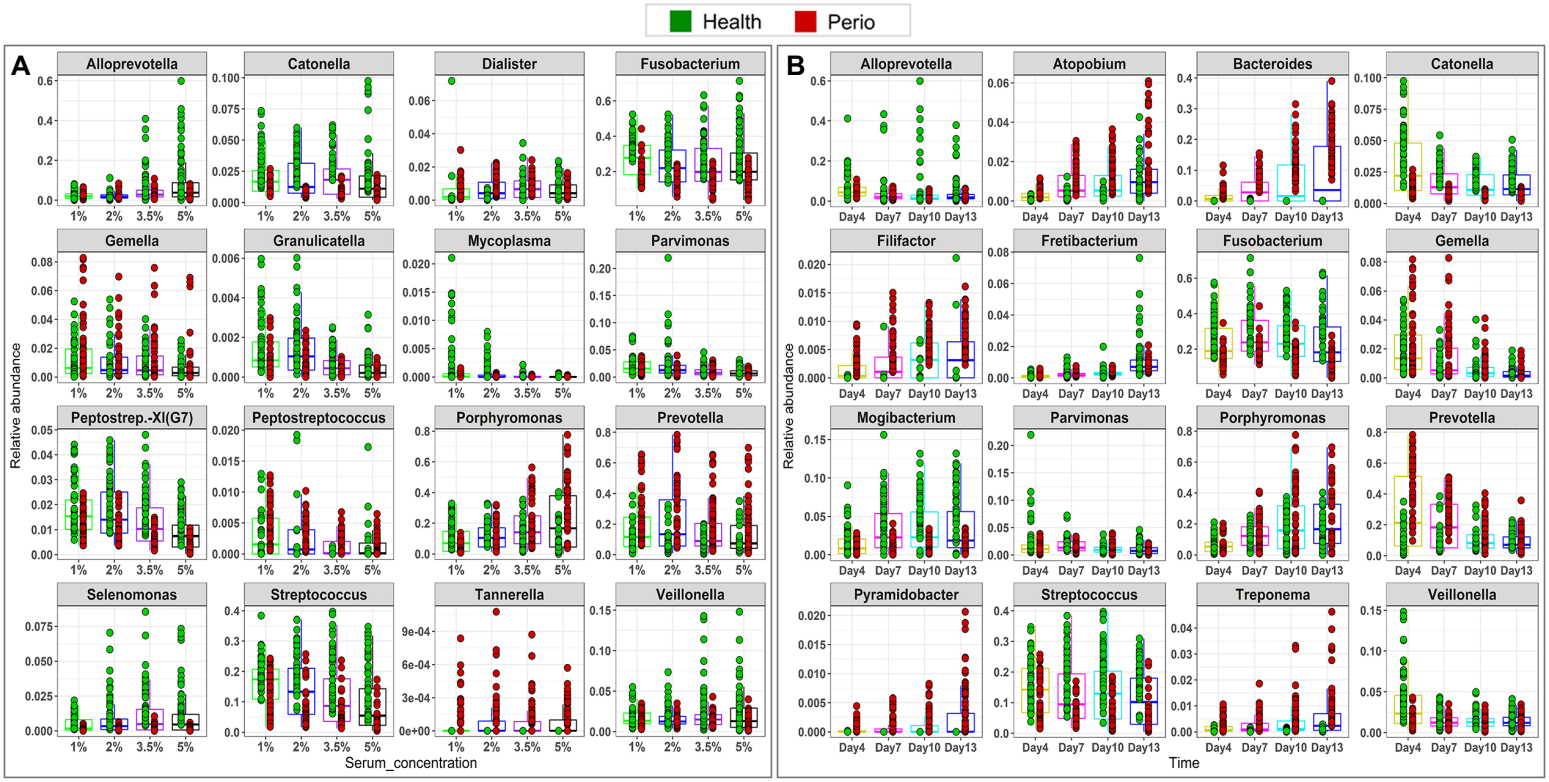
Selected differentially abundant genera by serum concentration and incubation period. Centered log-ratio (CLR) transformed data were analyzed with MaAsLin2 including health status, biological replicate, medium, shaking, and incubation time as fixed effects and technical replicates as random effects. Relative abundances are shown for 16 genera that significantly differed (FDR value < 0.1) by serum concentration **(A)** and 16 genera that differed by incubation period **(B)**. Plots were produced with R.

**Figure 6:**
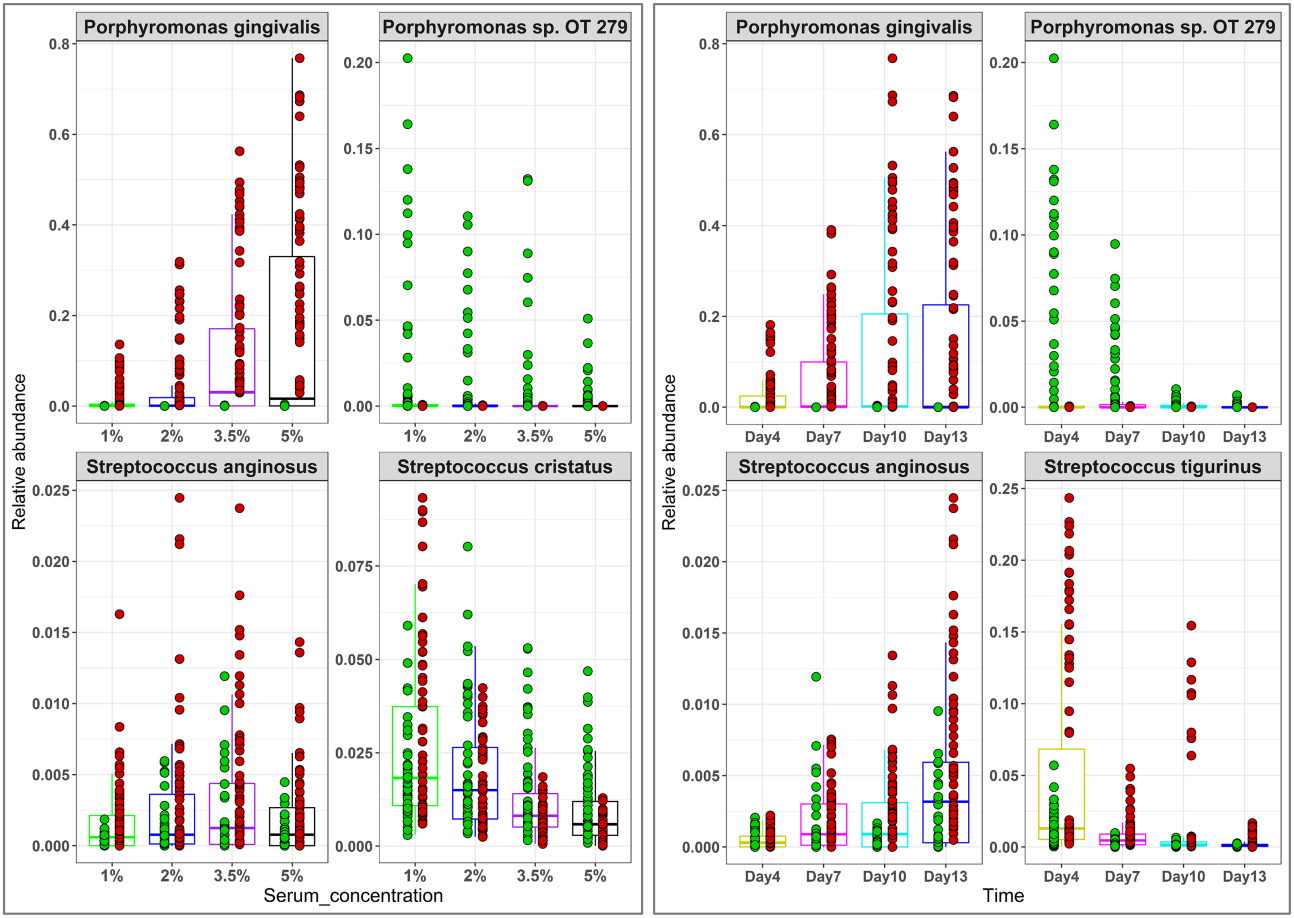
Selected differentially abundant species by serum concentration and incubation period. Centered log-ratio (CLR) transformed data were analyzed with MaAsLin2 including health status, biological replicate, medium, shaking, and incubation time as fixed effects and technical replicates as random effects. Relative abundances are shown for 4 pairs of ‘sister” species that significantly differed (FDR value < 0.1) between the two groups. Plots were produced with R.

The relative abundances of phyla and genera that significantly changed as a function of time and serum concentration are shown in Supplementary Figure 4 and Figure 5, respectively. At the phylum level, serum resulted in a dose-dependent increase in Bacteroidetes at the expense of Firmicutes and Fusobacteria, while a prolonged growth period was associated with an increase of Actinomyces and Spirochetes and slight decrease in Firmicutes, Fusobacteria and Bacteroidetes. At the genus-level, the major changes included substantial enrichment of *Porphyromonas* and *Alloprevotella* as a function of serum concentration at the expense of *Streptococcus*, *Fusobacterium* and *Prevotella*, and an increase in *Porphyromonas*, *Bacteriodes*, and *Mogibacterium* accompanied by a decrease in *Prevotella*, *Catonella*, and *Gemella* as a function of time. Figure 6 presents selected sister species that responded in opposite directions to increased serum concentration and incubation period.

### Shaking had limited effect on composition of the *in vitro* microbiomes

The independent effects of shaking on the growing microbiomes is shown in Supplementary Figure 5. Shaking increased biomass of the health-derived microbiome but not of the periodontitis-derived microbiomes. Statistically significant differences were observed for species richness and dysbiosis, but the magnitude of change was minor. Namely, shaking slightly increased in Chao index in the health-derived microbiomes and slightly decreased it in the periodontitis-derived microbiomes, while it marginally increased SMDI in both the health- and periodontitis-derived microbiomes, probably because of enrichment of genera *Treponema* and *Pyramidobacter* at the expense of *Gemella* and *Granulicatella* (Supplementary Figure 6). Shaking did not affect alpha diversity (Shannon index) in either microbiome type.

## Discussion

Periodontal diseases are associated with an increase in subgingival biomass and a shift in the overall balance of the composition of the biofilm (Curtis et al., 2000). The factors that drive the development of this dysbiotic community could include changes in the status of the host defenses but probably also include changes to the nutritional profile in the local environment to enable the fastidious, disease-associated microbes to be able to grow and to outcompete the species associated with health. While the primary purpose of the present study was to optimize conditions for modeling the subgingival microbiome, it also enabled us to assess the potential role of nutrients as drivers of subgingival microbial dysbiosis.

Saliva is a major nutritional source for oral micro-organisms, but the healthy gingival crevice is also exposed to small quantities of a serum-like exudate *via* the flow of gingival crevicular fluid (GCF) (Kilian et al., 2016). During periodontal inflammation, the flow of GCF is increased markedly. Apart from delivering components of the host defenses, this exudate is important nutritionally and is capable of supporting the growth of proteolytic and fastidious bacteria. Early studies demonstrated that growing subgingival microbiota in the presence of serum enriched for *Bacteriodes*, *Peptostreptococcus* and *Fusobacterium* (ter Steeg et al., 1987). Along the same lines, recent studies by our group and others have shown that adding serum to a medium results in significant enrichment of periodontal pathogens, including *P. gingivalis* (Cieplik et al., 2019; Naginyte et al., 2019; Baraniya et al., 2020). In our previous study (Baraniya et al., 2020), it was established that saliva with serum outperformed other nutrient-rich media for modeling subgingival biofilms, both from health and disease; nonetheless, serum, even at the lowest concentration (5%) still resulted in overgrowth of *P. gingivalis*, especially in the health-derived microbiomes.

Consequently, in this study we used lower concentrations of serum in saliva, and also varied the length of incubation and evaluated the impact of shaking during biofilm development. Composition of the *in vitro* microbiomes were quantified in far greater detail and at a finer resolution than our previous study. The major findings of the study are as follows. First, the study demonstrates the reliability of the model to reproducibly generate normobiotic and dysbiotic subgingival microbiomes *in vitro.* Second, the health-derived and periodontitis-derived microbiomes have different requirements for optimizing their community structure. Overall, and taking into consideration species richness, alpha and beta diversity and SMDI values, the health-derived microbiomes grown for 4 days at 1% serum were closest to the health inoculum, while for periodontitis, the microbiomes grown for 7 days at 35–5% serum, or for 10 days at 1%–2% serum, were comparable and the most similar to the respective inoculum. The third important finding is that serum and time independently and differently contributed to dysbiosis. For example, while *P. gingivalis* increased as a function of both factors, *Treponema*, *Fretibacterium*, and *Pyramidobacter* increased primarily as a function of time, while *Tannerella* and *Selenomonas* increased with serum concentration. There were taxa that changed in opposite directions. For examples, *Veillonella* increased with serum concentration but decreased with time.

It is important to note that the increase in SMDI was relative within each microbiome type, i.e., the microbiomes still separated by type regardless of time and serum concentration (Figure 2A). In other words, the composition of the original inoculum was the primary determinant of the composition and level of dysbiosis of the respective *in vitro* microbiomes. While there was an overlap in SMDI values between health-derived microbiome grown at 3.5%–5% serum for 13 days and periodontitis-derived microbiomes grown at 1%–2% for 4 days, it is unlikely that the former would fully assimilate into periodontitis-associated microbiomes if grown for longer periods or higher serum concentrations since some key periodontal pathogens were missing, e.g., *P. gingivalis.*

Apart from dysbiosis, the temporal microbial changes observed in the *in vitro* microbiomes are consistent with those reported for *in vivo* biofilms. For instance, during the early stages of oral biofilm development, *Firmicutes* including *Streptococcus* and *Veillonella* are primary colonizers (Klug et al., 2016). Other bacteria actively involved in early stages include *Actinomyces*, *Gemella*, *Granulicatella*, *Neisseria*, *Prevotella*, and *Rothia* (Diaz et al., 2006). In this study, these early colonizers were significantly higher in abundance on Day 4 than at later time points. *Fusobacterium* species are considered as “bridge organisms” between early and late colonizers, which facilitates the growth of late colonizers by creating a conducive environment using its ability to coaggregate with most other species (Sharma et al., 2005; Aruni et al., 2015). Here, *Fusobacterium* increased during early biofilm development, though this was followed by a decrease in abundance over time. Late colonizers include many periodontal pathogens such as *Porphyromonas*, *Tannerella*, and *Treponema* (Aruni et al., 2015), which in our study were significantly more abundant on days 10 and 13 relative to days 4 and 7.

As expected, and although the model captured much of the species richness and diversity of the clinical inocula, there were a few species which did not thrive and, as discussed above, some species which were enriched in the *in vitro* biofilms, and this implies that there are other factors that determine the composition of the subgingival microbiome *in vivo*. This is a limitation of our (and of others) model (Brown et al., 2019), but the system described here also provides the opportunity to further explore these factors including components of the host defenses. For example, it would be interesting to use native rather than heat-inactivated serum to assess how activation of the complement system affects the model. Antibodies may also have a role is shaping the microbiome, so it is important to account for individual variations in antibodies, for example by comparing different batches of human serum. Nevertheless, and despite these limitations, the close similarity of the developing biofilms to the microbiomes reported *in vivo* in health and disease means that the model could be used to investigate potential interventions to prevent or reverse dysbiosis.

In conclusion, this study demonstrates that saliva supplemented with heat-inactivated serum can be used to reliably and reproducibly grow normobiotic and dysbiotic subgingival microbiomes from clinical samples. However, serum and incubation periods need to be adjusted differently for the health- and periodontitis-derived microbiomes to maximize their similarity to *in vivo* biofilms. The study also shows that time and serum are independent drivers of dysbiosis. This model is an easy and effective system to study subgingival biofilm colonization in disease and health, and to evaluate interventions to prevent or reverse dysbiosis.

## Data availability statement

The datasets presented in this study can be found in online repositories. The names of the repository/repositories and accession number(s) can be found at: NCBI—PRJNA881921.

## Ethics statement

The studies involving human participants were reviewed and approved by Temple University’s Institutional Review Board under protocol no. 25586. The patients/participants provided their written informed consent to participate in this study.

## Author contributions

DB contributed to design, the data acquisition, analysis, and interpretation and drafted the manuscript. TC contributed to the data analysis and critically revised the manuscript. JA and SC contributed to the data acquisition and critically revised the manuscript. TD, DD, and PM contributed to the design and data interpretation and critically revised the manuscript. NA conceived the study, contributed to the design, data analysis, and interpretation, and critically revised the manuscript. All authors contributed to the article and approved the submitted version.

## Funding

This study was supported by the National Institute of Dental and Craniofacial Research (grant 1R03DE028379). Publication of this article was funded in part by the Temple University Libraries Open Access Publishing Fund and in part by Cary R. Klimen Oral Health Sciences Research Program Fund.

## Conflict of interest

The authors declare that the research was conducted in the absence of any commercial or financial relationships that could be construed as a potential conflict of interest.

## Publisher’s note

All claims expressed in this article are solely those of the authors and do not necessarily represent those of their affiliated organizations, or those of the publisher, the editors and the reviewers. Any product that may be evaluated in this article, or claim that may be made by its manufacturer, is not guaranteed or endorsed by the publisher.

## Supplementary material

The Supplementary material for this article can be found online at: https://www.frontiersin.org/articles/10.3389/fmicb.2022.1031029/full#supplementary-material
