# Supplementary material for: Optimization of conditions for in vitro modeling of subgingival normobiosis and dysbiosis
Source: Front Microbiol. 2022 Nov 3;13:1031029. doi: 10.3389/fmicb.2022.1031029 (PMC9670125; doi:10.3389/fmicb.2022.1031029)
Supplement: Supplementary file 1 [file Table_1.DOCX]

**Supplementary Table 1.** Demographic information of subgingival plaque donors.

| Experiment 1 | Periodontitis | Healthy |
| --- | --- | --- |
| Age (mean±SD) | 49.2±10.05 years | 36.8± 21.39 years |
| Age range | 40-65 years | 23-74 years |
| Gender* | 1M/4F | 2M/3F |
| Ethnicity^#^ | 2A/2AA/1H | 1A/2AA/2C |

| Experiment 2 | Periodontitis | Healthy |
| --- | --- | --- |
| Age (mean±SD) | 63.6±7.26 years | 32.4±13.99 years |
| Age range | 57-72 years | 23-57 years |
| Gender* | 2 M/3 F | 4 M/1 F |
| Ethnicity^#^ | 3AA/1C/1H | 4C/1H |

*Gender: M – Male, F- Female

^#^Ethnicity: A- Asian, AA-African American, C-Caucasian, H-Hispanic
