# Supplementary material for: Optimization of conditions for in vitro modeling of subgingival normobiosis and dysbiosis
Source: Front Microbiol. 2022 Nov 3;13:1031029. doi: 10.3389/fmicb.2022.1031029 (PMC9670125; doi:10.3389/fmicb.2022.1031029)
Supplement: Supplementary file 2 [file Presentation_1.PPTX]

## Slide 1
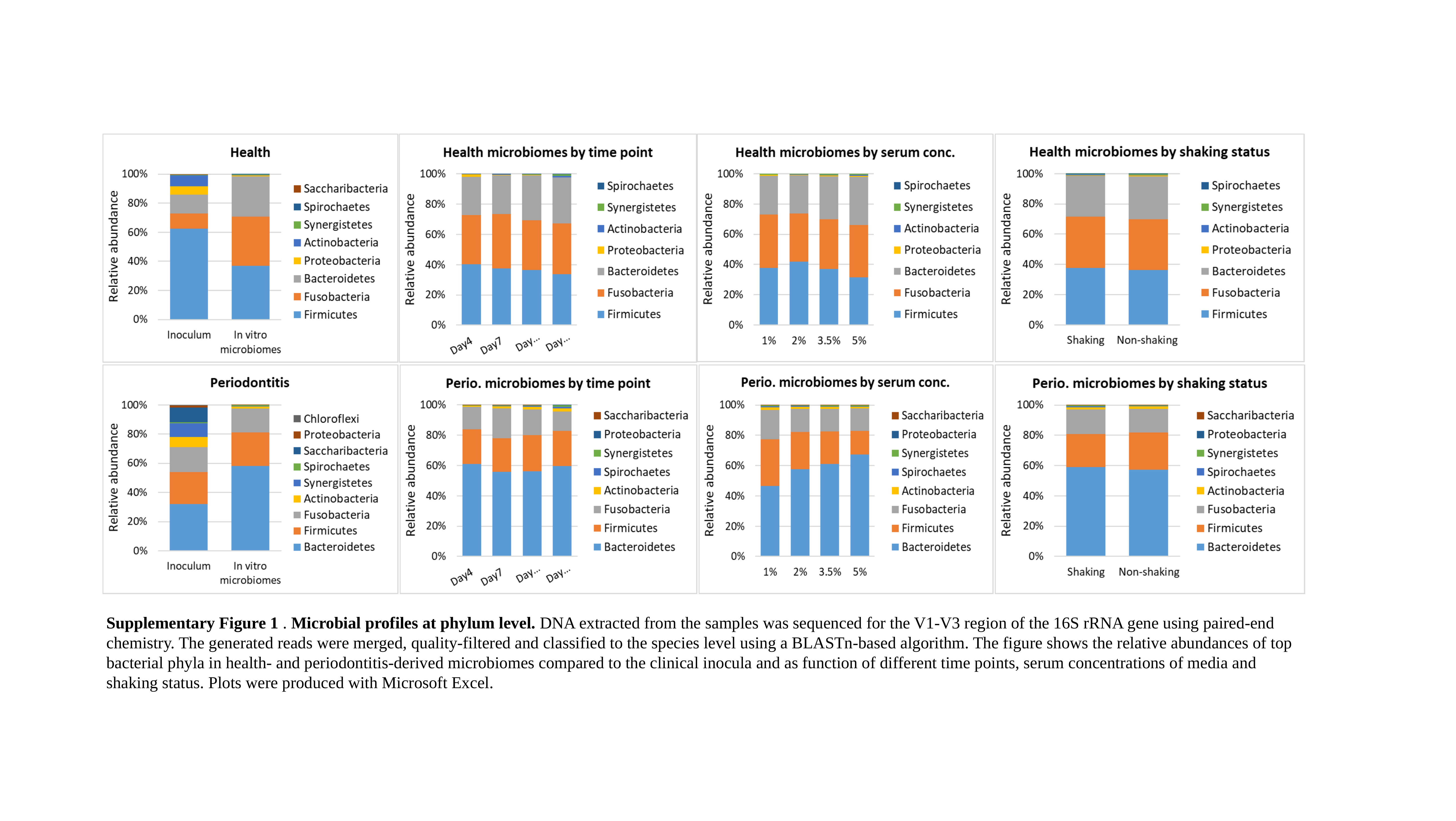

Supplementary Figure 1 . Microbial profiles at phylum level. DNA extracted from the samples was sequenced for the V1-V3 region of the 16S rRNA gene using paired-end chemistry. The generated reads were merged, quality-filtered and classified to the species level using a BLASTn-based algorithm. The figure shows the relative abundances of top bacterial phyla in health- and periodontitis-derived microbiomes compared to the clinical inocula and as function of different time points, serum concentrations of media and shaking status. Plots were produced with Microsoft Excel.

## Slide 2
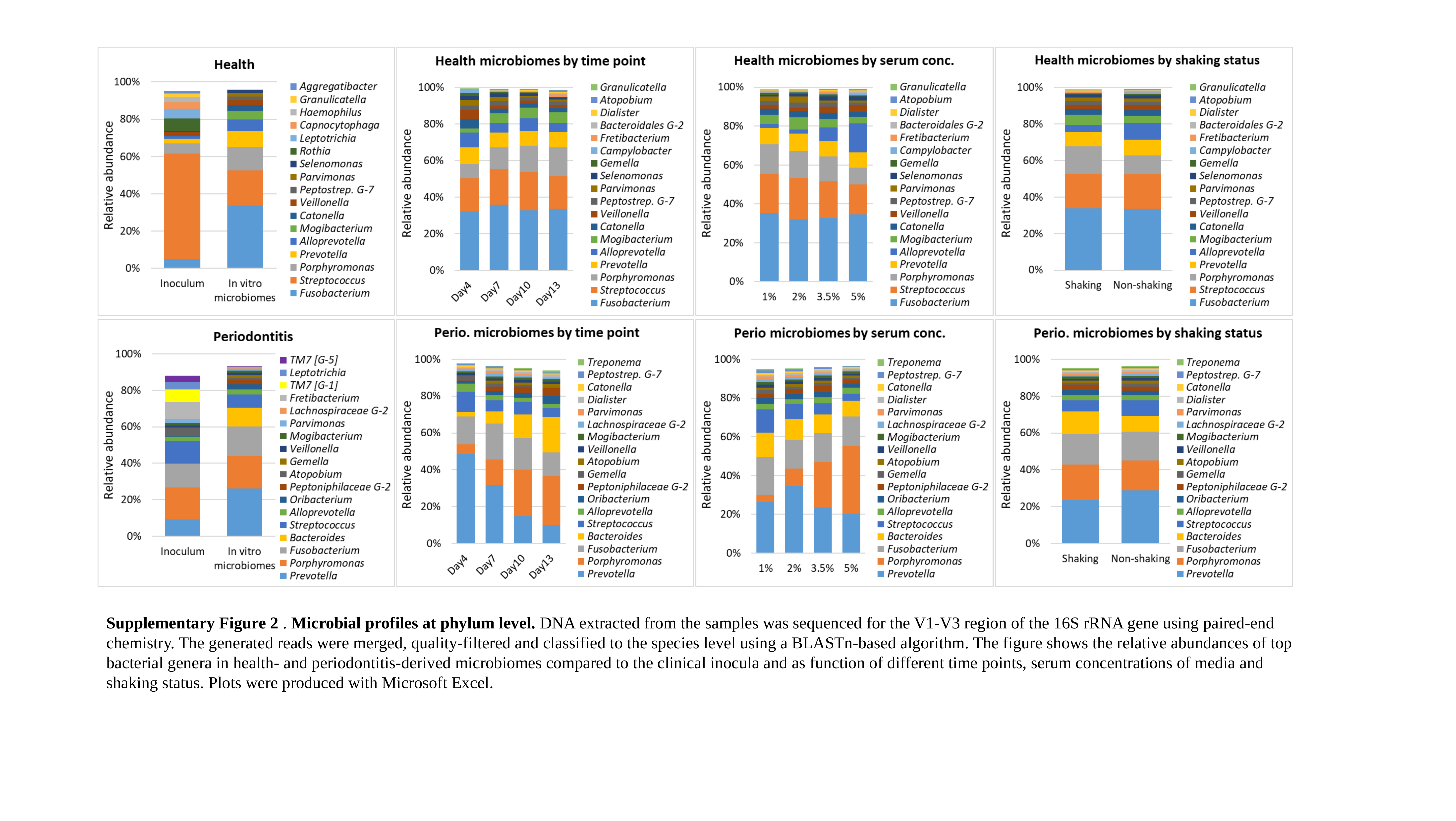

Supplementary Figure 2 . Microbial profiles at phylum level. DNA extracted from the samples was sequenced for the V1-V3 region of the 16S rRNA gene using paired-end chemistry. The generated reads were merged, quality-filtered and classified to the species level using a BLASTn-based algorithm. The figure shows the relative abundances of top bacterial genera in health- and periodontitis-derived microbiomes compared to the clinical inocula and as function of different time points, serum concentrations of media and shaking status. Plots were produced with Microsoft Excel.

## Slide 3
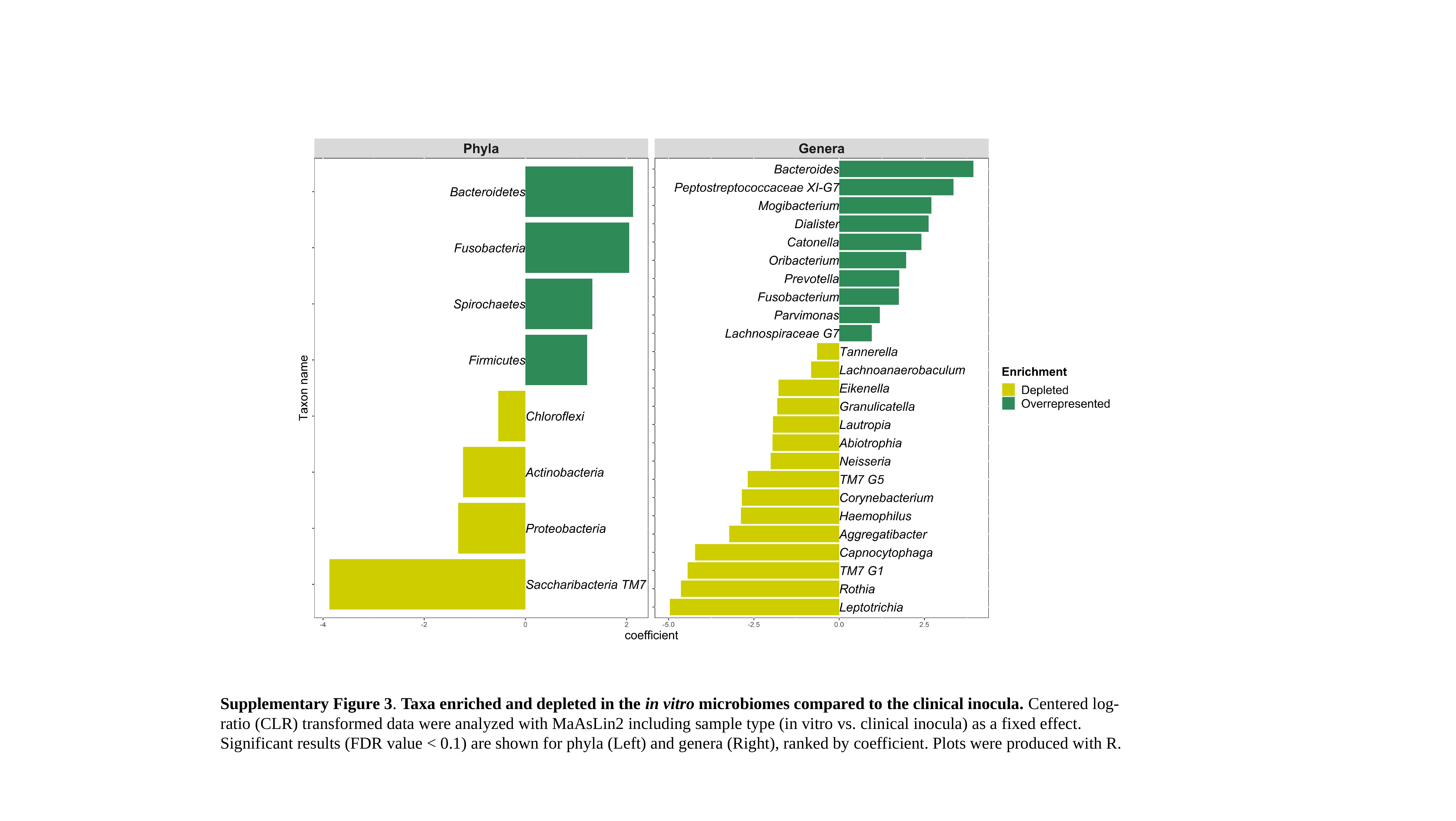

Supplementary Figure 3. Taxa enriched and depleted in the in vitro microbiomes compared to the clinical inocula. Centered log-ratio (CLR) transformed data were analyzed with MaAsLin2 including sample type (in vitro vs. clinical inocula) as a fixed effect. Significant results (FDR value < 0.1) are shown for phyla (Left) and genera (Right), ranked by coefficient. Plots were produced with R.

## Slide 4
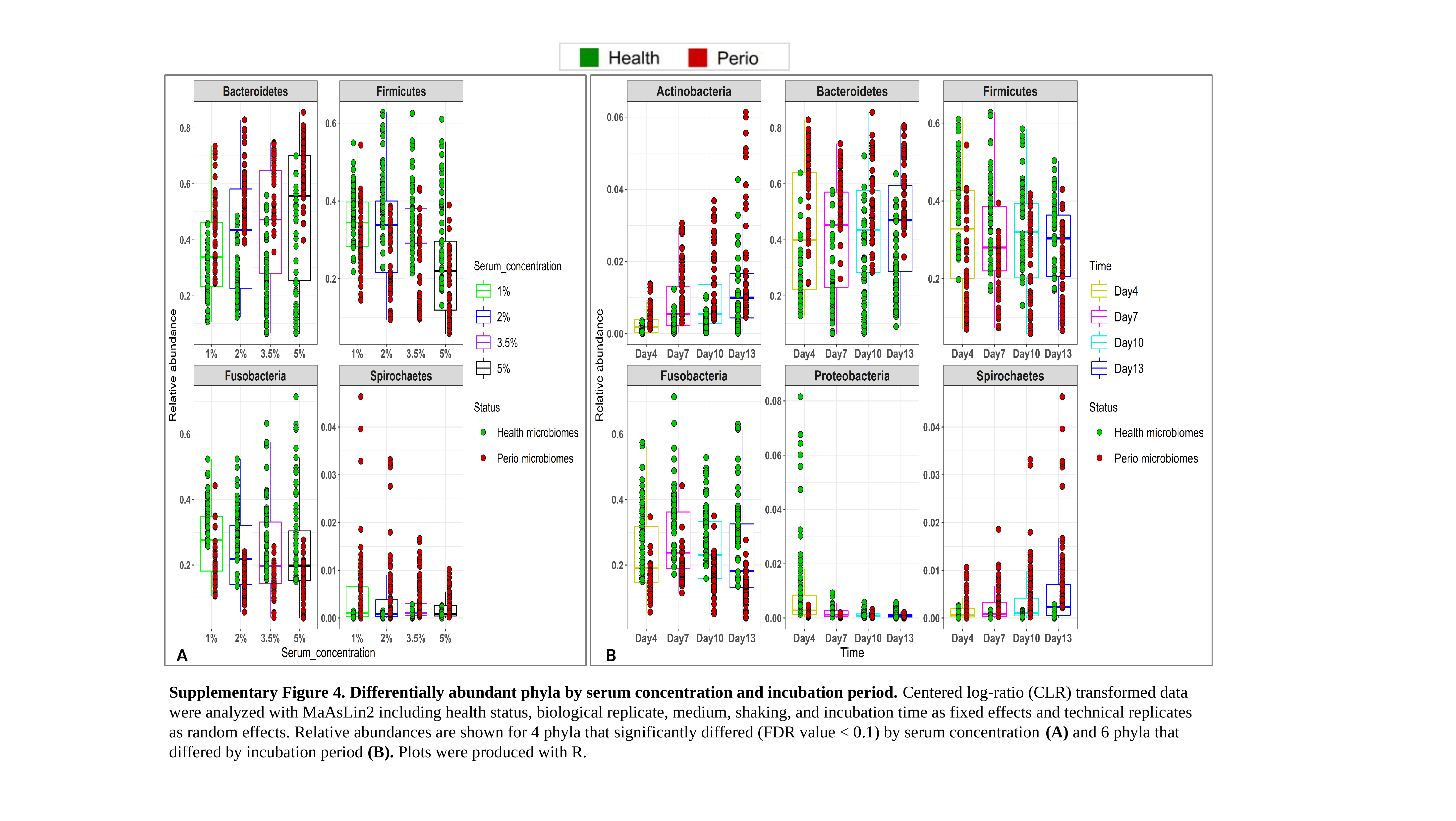

A B
Supplementary Figure 4. Differentially abundant phyla by serum concentration and incubation period. Centered log-ratio (CLR) transformed data were analyzed with MaAsLin2 including health status, biological replicate, medium, shaking, and incubation time as fixed effects and technical replicates as random effects. Relative abundances are shown for 4 phyla that significantly differed (FDR value < 0.1) by serum concentration (A) and 6 phyla that differed by incubation period (B). Plots were produced with R.

## Slide 5
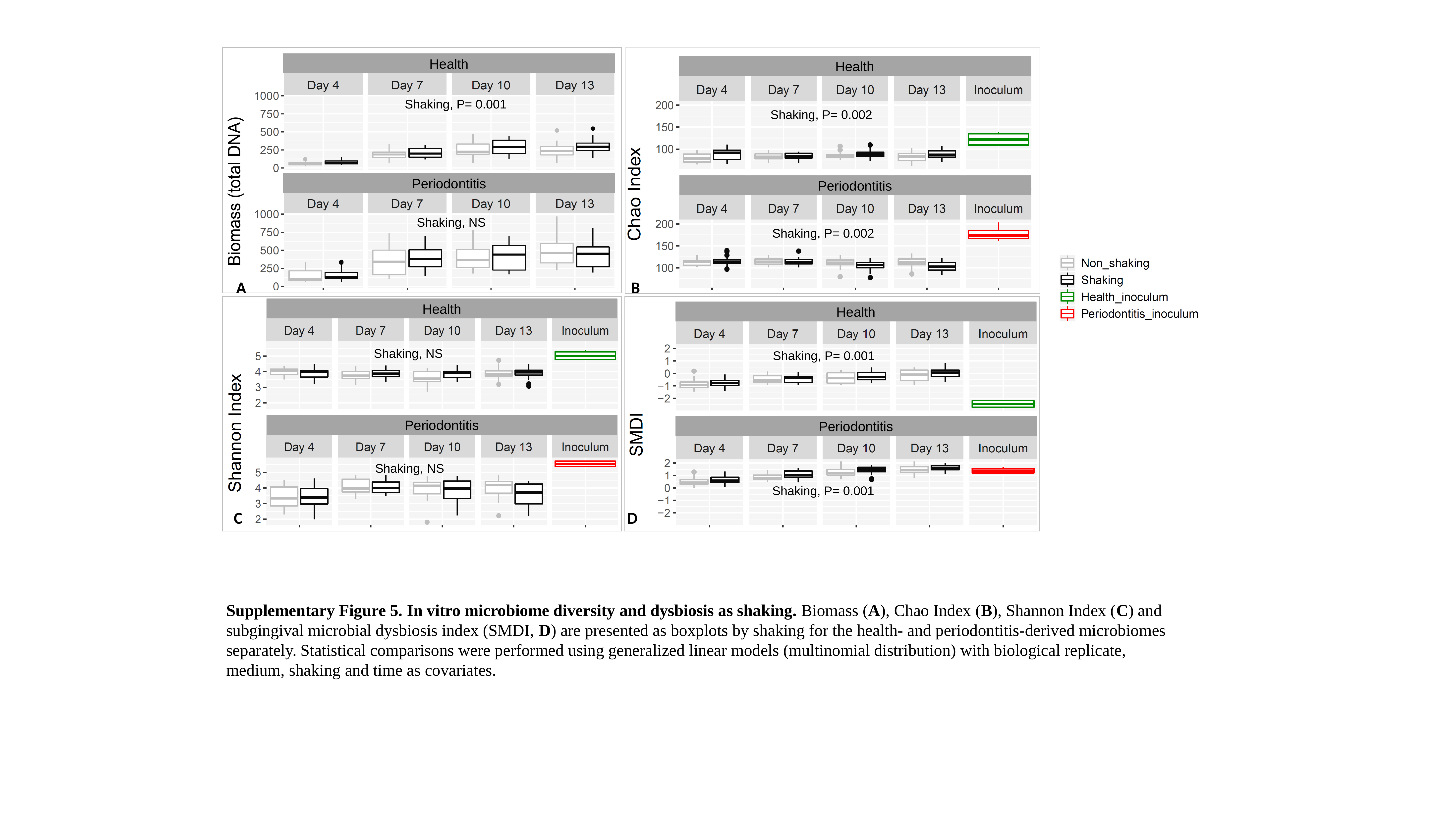

Health
Health
Shaking, P= 0.001
Shaking, P= 0.002
Periodontitis
Periodontitis
Shaking, NS
Shaking, P= 0.002
A B
Health
Health
Shaking, NS
Shaking, P= 0.001
Periodontitis
Periodontitis
Shaking, NS
Shaking, P= 0.001
C D
Supplementary Figure 5. In vitro microbiome diversity and dysbiosis as shaking. Biomass (A), Chao Index (B), Shannon Index (C) and subgingival microbial dysbiosis index (SMDI, D) are presented as boxplots by shaking for the health- and periodontitis-derived microbiomes separately. Statistical comparisons were performed using generalized linear models (multinomial distribution) with biological replicate, medium, shaking and time as covariates.

## Slide 6
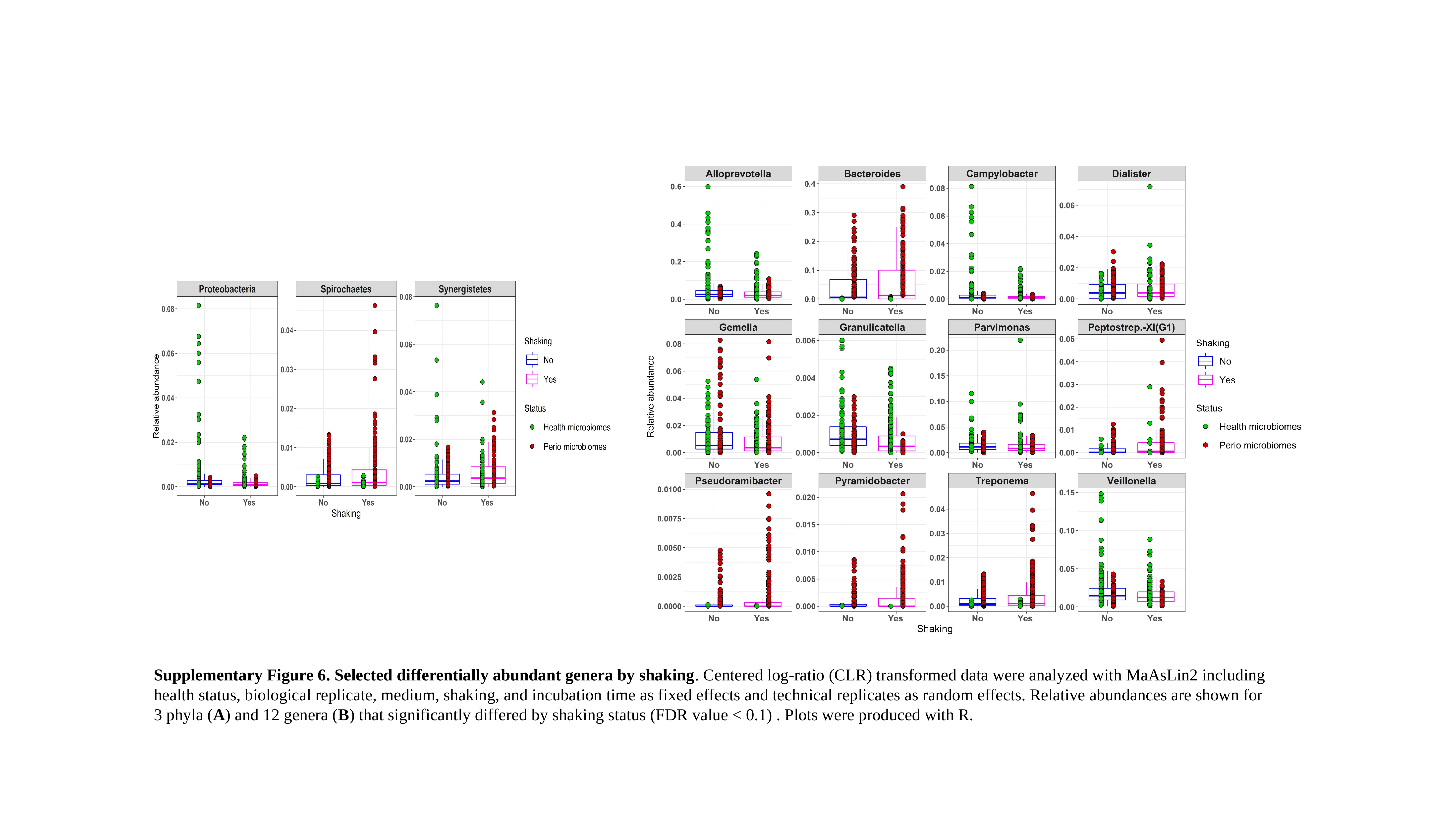

Supplementary Figure 6. Selected differentially abundant genera by shaking. Centered log-ratio (CLR) transformed data were analyzed with MaAsLin2 including health status, biological replicate, medium, shaking, and incubation time as fixed effects and technical replicates as random effects. Relative abundances are shown for 3 phyla (A) and 12 genera (B) that significantly differed by shaking status (FDR value < 0.1) . Plots were produced with R.
